# Cancer-associated fibroblasts enhance metastatic potential of lung cancer cells through IL-6/STAT3 signaling pathway

**DOI:** 10.18632/oncotarget.18814

**Published:** 2017-06-28

**Authors:** Limin Wang, Limin Cao, Huimin Wang, Boning Liu, Qicheng Zhang, Zhaowei Meng, Xiang Wu, Qinghua Zhou, Ke Xu

**Affiliations:** ^1^ Tianjin Key Laboratory of Lung Cancer Metastasis and Tumor Microenviroment, Tianjin Lung Cancer Institute, Tianjin Medical University General Hospital, Tianjin 300052, China; ^2^ Department of Nuclear Medicine, Tianjin Medical University General Hospital, Tianjin 300052, China; ^3^ Core Facility Center, Tianjin Medical University General Hospital, Tianjin 300052, China

**Keywords:** lung cancer, cancer-associated fibroblasts, metastasis, EMT, IL-6

## Abstract

Recent studies indicate that cancer-associated fibroblasts (CAFs) are involved in tumor growth, invasion and metastasis, however, the underling mechanisms remain unclear. In the present study, we investigated the role of CAFs on the metastatic potential of lung cancer cells. The stromal fibroblasts we isolated from lung cancer tissues presented CAFs characteristics with high levels of α-smooth muscle actin (α-SMA) and fibroblast-activating protein (FAP). Our data showed that the conditioned medium from cultured CAFs (CAF-CM) dramatically enhanced migration and invasion of lung cancer cells. CAF-CM induced epithelial-mesenchymal transition (EMT) by regulating the expression of EMT-associated markers E-cadherin and vimentin, and also modulated metastasis-related genes MMP-2 and VEGF both *in vitro* and *in vivo*. Further mechanistic studies demonstrated that CAFs enhanced the metastatic potential of lung cancer cells by secreting IL-6, subsequently activating of JAK2/STAT3 signaling pathway. Additionally, the inhibition of IL-6/STAT3 signaling pathway by IL-6 neutralizing antibody or specific inhibitors of JAK2/STAT3 reversed CAF-CM induced EMT and migration of lung cancer cells. Taken together, these findings revealed a novel mechanism that CAFs induced EMT and promoted metastasis of lung cancer cells through the IL-6/STAT3 signaling pathway.

## INTRODUCTION

Lung cancer is the most frequently diagnosed cancer and the leading cause of cancer death among males and the second leading cause of cancer death among females. It is estimated that there were 1.8 million new cases and 1.6 million deaths annually, accounting for about 19% of all cancer deaths [1]. The majority of lung cancer is non-small cell lung cancer (NSCLC), which comprises approximately 85% of total lung cancer. The overall 5-year survival rate of this disease is 15% [2]. Nearly 90% of lung cancer patients die of metastasis, therefore, a better understanding of the mechanism of lung cancer metastasis is urgently needed.

Tumor metastasis is a complicated biological process, including intravasation, extravasation, colonization, and many genes and signaling pathways are involved [3]. Tumors consist of malignant cancer cells and stromal cells which constitute the tumor microenvironment. Numerous studies showed that the metastasis potential of cancer cells intensively depends on the tumor microenvironment [4]. Cancer-associated fibroblasts (CAFs) are one of the major components in the tumor stroma, they display a specific subset of markers, including α-smooth muscle actin (α-SMA), fibroblast-activating protein (FAP), fibroblast-specific protein-1 (FSP1), tenascin C, and neural-glial antigen [5].

Tumor cells may activate stromal fibroblast cells into CAFs by the stimulation of paracrine growth factor [6, 7]. Transforming growth factor-β (TGFβ) actives and recruits fibroblast in several types of cancer [8, 9], and platelet-derived growth factor (PDGF) secreted by cancer cells can activate and induce the proliferation of fibroblasts [10]. On the other hand, CAFs support tumorigenesis by stimulating angiogenesis, cancer cell proliferation, and invasion [6]. CAFs promote cancer cell growth and metastasis by secreting various growth factors and cytokines, including TGFβ [11], stromal cell-derived factor-1 (SDF-1) [12], CXCL1 [13], tumor necrosis factor-α (TNFα) [14], as well as microRNAs [15, 16] and exosome [17, 18].

Currently, few studies focused on the role of CAFs on lung cancer cell growth and metastasis, and the interaction between CAFs and lung cancer cells has not been elucidated. In this study, we have explored the influence of CAFs on the invasion and metastasis of lung cancer cells. The mechanism study showed that IL-6/STAT3 signaling pathway mediated the enhanced effect of CAFs on lung cancer cell metastasis.

## RESULTS

### The characterization of CAFs and NFs isolated from lung cancer tissues

**Figure 1:**
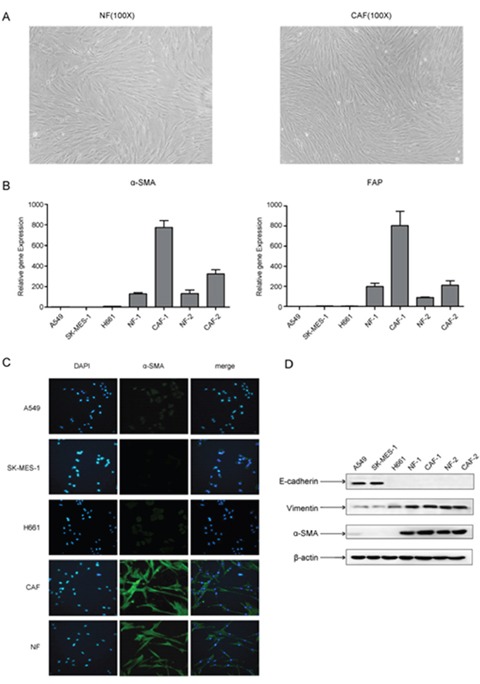
Characteristics of CAFs and NFs isolated from lung cancer tissues **(A)** Morphological features of primary cultured NFs and CAFs (×100 magnification). **(B)** The mRNA expression levels of α-SMA and FAP were detected by qPCR. **(C)** The expression of α-SMA was detected by immunofluorescence staining. **(D)** The expression of E-cadherin, vimentin and α-SMA were detected by Western blot.

In order to investigate the effect of primary cultured fibroblast on the metastasis potential of lung cancer cells, we isolated 2 pairs of CAFs and normal fibroblasts (NFs) from primary tumor tissues and matched adjacent normal lung tissues from 2 lung cancer patients. After passaging the primary cultured fibroblasts, both CAFs and NFs displayed a thin and spindle-like appearance, suggesting that these cells were fibroblasts (Figure 1A). However, there was no significant difference between CAFs and NFs in morphology. We then examined the mRNA expression of the activated myofibroblast marker α-SMA and FAP in CAFs, NFs and lung cancer cell line A549 and SK-MES-1 cells by qPCR. CAFs and NFs expressed higher mRNA level of α-SMA and FAP than lung cancer cells, and as expected, α-SMA and FAP expression level were higher in CAFs than in NFs (Figure 1B). We further detected the protein level of α-SMA, the mesenchymal marker vimentin, and the epithelial marker E-cadherin. Both CAFs and NFs expressed high level of α-SMA and vimentin, did not express E-cadherin. Whereas, lung cancer A549 and SK-MES-1 cells expressed high level of E-cadherin, and low level of α-SMA and vimentin (Figure 1C, 1D). Taken together, these results suggest that primary cultured fibroblasts derived from patients with lung cancer retain the features of CAFs and NFs.

### CAFs enhanced the migration and invasion of lung cancer cells

**Figure 2:**
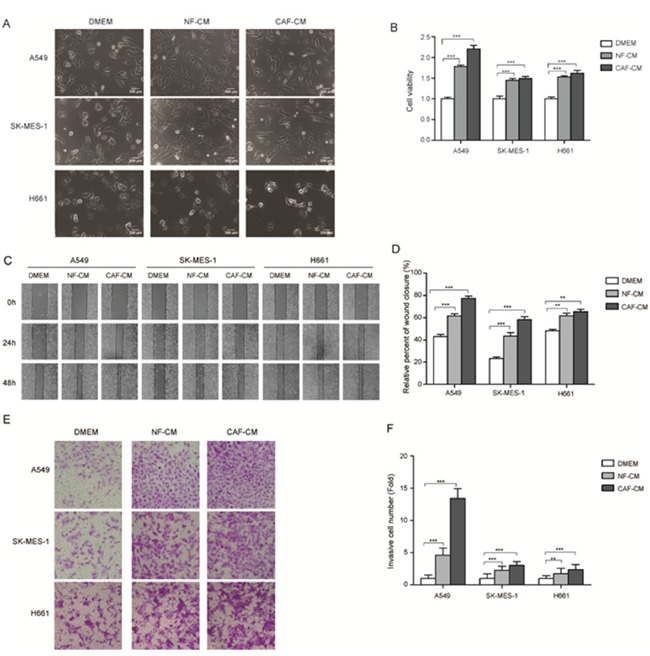
The stromal fibroblasts cultured medium enhanced the abilities of migration and invasion of lung cancer cells **(A)** Morphological characters of lung cancer cells cultured with CAF-CM and NF-CM. **(B)** CAF-CM and NF-CM stimulated the growth of A549 and SK-MES-1 cells. Lung cancer cells were cultured in DMEM or DMEM mixed with NF-CM or CAF-CM (1:1) for 72 h. Cell growth were measured by CCK-8. **(C)** Wound healing assays were performed to evaluate cell migration ability after 48 h, representative photographs are presented (×40 magnification) **(D)** Relative adhension rates were presented. **(E)** Transwell assays were performed to evaluate cell invasion ability after 48h, representative photographs are presented (×200 magnification). **(F)** Relative invasive cell number was presented. Values represent the mean ± SD of three independent experiments. ***P < 0.001.

Lung cancer cell lines we studied were A549 cells (adenocarcinoma) and SK-MES-1 cells (squamous cell carcinoma), which represented different pathological subtypes of lung cancer. CAF-CM or NF-CM was collected after 48 h culture of CAFs or NFs in DMEM/F12 (1:1) medium (GIBCO). We first evaluated the effect of CAF-CM and NF-CM on the growth of lung cancer cells. After treated with CAF-CM and NF-CM, lung cancer cells became more spindle shaped (Figure 2A). Figure 2B showed that both CAF-CM and NF-CM stimulated lung cancer cell proliferation, and CAF-CM was more effective on A549 cells.

To investigate the effect of CAFs on cell migration, A549 and SK-MES-1 cells were pretreated with CAF-CM or NF-CM for 48 h, DMEM medium was used as control, then wound healing assay were performed. The adhesive rate for A549 and SK-MES-1 cells cultured with control medium were 42.9% and 23.2%, respectively. When cultured with NF-CM the adhesive rate were increased to 61.6% and 43.5%, respectively; when cultured with CAF-CM, the adhesive rate were increased more dramatically, to 77.3% and 58.3%, respectively (Figure 2C, 2D). The effect of CAF-CM on cell invasion was examined by trans-well assay. Figure 2E and 2F showed that both CAF-CM and NF-CM boosted the invasion ability of lung cancer cells compared with control medium, and CAF-CM were more effective. Taken together, our data indicated that CAF-CM enhanced the migration and invasion ability of lung cancer cells.

### CAFs induced EMT in lung cancer cells and regulated metastasis-related genes

**Figure 3:**
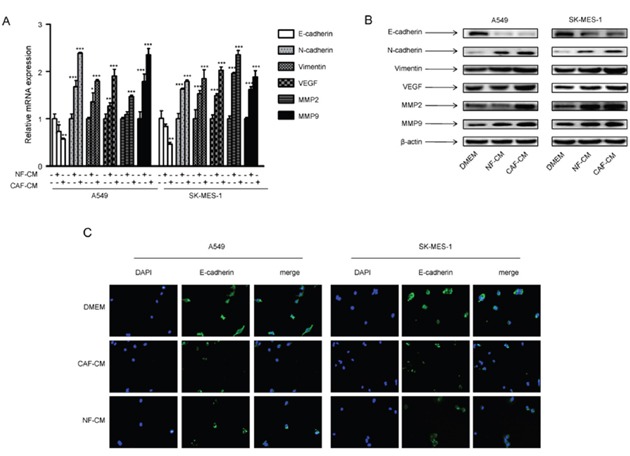
CAF-CM induced EMT phenotype and regulated metastasis-related genes in lung cancer cells A549 and SK-MES-1 cells were incubated with CAF-CM or NF-CAM for 24 h. **(A)** The mRNA expression levels were detected by qPCR. **(B)** The protein expression levels were detected by Western blot assay. **(C)** The expression of E-cadherin was detected by immunostaining. Values represent the mean ± SD of three independent experiments. *P<0.05; **P<0.01; ***P<0.001.

As EMT plays a pivotal role in tumor metastasis, we investigated the changes of EMT phenotype of lung cancer cells by CAFs. We detected the expression of epithelial marker E-cadherin and mesenchymal marker vimentin. E-cadherin expression was decreased by CAF-CM and NF-CM in A549 cells and SK-MES-1 cells, whereas, vimentin expression was increased at both mRNA and protein level (Figure 3A, 3B). By immunostaining, we found that both membranous and cytoplasmic expressions of E-cadherin were decreased (Figure 3C).

We further studied the effect of CAFs on metastasis-related genes vascular endothelial growth factor (VEGF) and metalloproteinase 2 (MMP2). Our data showed that CAF-CM and NF-CM upregulated mRNA and protein expression of VEGF and MMP2, and CAFs had stronger effect (Figure 3A, 3B). These results demonstrated that CAFs enhanced metastasis potential of lung cancer cells by inducing EMT programming and modulating metastasis-related genes.

### IL-6 mediated the effect of CAFs on metastasis potential of lung cancer cells

**Figure 4:**
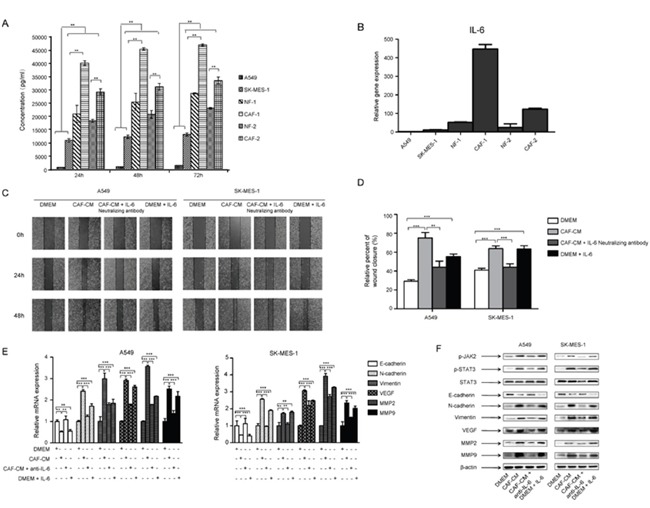
IL-6 mediated the effect of CAFs on migration and EMT in lung cancer cells **(A)** CAF-CM, NF-CM and lung cancer cell lines cultured medium were collected. The IL-6 levels were quantified by ELISA. **(B)** The mRNA expression of IL-6 in CAFs, NFs and lung cancer cell lines were detected by qPCR. **(C)** Lung cancer cells were treated with DMEM, CAF-CM, CAF-CM + IL-6 neutralizing antibody (5 μg/ml) or DMEM + IL-6 (200 ng/ml) for 24 to 48 h. Cell migration ability was measured by wound healing assays. **(D)** Relative adhension rates at 48 h time point were presented. **(E)** Lung cancer cells were cultured with DMEM, CAF-CM, CAF-CM + IL-6 neutralizing antibody (5 μg/ml) or DMEM + IL-6 (200 ng/ml) for 24 h. mRNA expression were detected by qPCR. **(F)** Lung cancer cells were treated with DMEM, CAF-CM, CAF-CM + IL-6 neutralizing antibody (5 μg/ml) or DMEM + IL-6 (200 ng/ml) for 24 h. Protein expression were detected by Western blot. Values represent the mean ± SD from three independent experiments. **P<0.01; ***P<0.001.

CAF-CM enhanced migration and invasion of lung cancer cells, suggested that CAF may secrete proteins to change EMT status and promote lung cancer cell metastasis. IL-6 is mainly produced by activated T cells and fibroblasts, and it induces EMT in many types of cancer cells. We therefore examined whether CAFs secreted any IL-6 into CAF-CM. First we detected the endogenous IL-6 level in CAFs and lung cancer cells. CAFs and NFs expressed significant higher level of IL-6 than lung cancer cells (Figure 4A). Then we examined the IL-6 level in culture medium at indicated time points by ELISA. Figure 4B showed that IL-6 levels in CAF-CM and NF-CM were higher than in lung cancer cell culture medium, which were consistent with endogenous IL-6 levels in cells.

To investigate whether IL-6 plays a role in CAFs mediated metastasis, we added 200 ng/ml of human recombinant IL-6 to normal medium cultured A549 and SK-MES-1 cells, or 5 μg/ml of IL-6 neutralizing antibody to CAF-CM cultured A549 and SK-MES-1 cells, and analyzed by wound healing assay. As shown in Figure 4C and 4D, IL-6 enhanced the migration of lung cancer cells, and IL-6 neutralizing antibody abolished the effect of CAF-CM on cell migration. We next investigated the effect of IL-6 on EMT status and metastasis-related genes in lung cancer cells. IL-6 decreased the expression of E-cadherin, whereas increased the expression of vimentin, N-cadherin, MMP2, MMP9 and VEGF at both mRNA and protein levels. Further studies showed that IL-6 neutralizing antibody reversed this effect (Figure 4E, 4F). Taken together, these results demonstrated that CAFs induced EMT and enhanced the metastasis potential of lung cancer cells by secreting IL-6.

### CAFs activated JAK2/STAT3 signaling pathway in lung cancer cells via CAF-secreted IL-6

Numerous studies show that IL-6/STAT3 signaling pathway plays an important role in tumor metastasis. To explore the mechanism underlying the role of CAFs on tumor metastasis, we investigated the effect of CAFs on IL-6/STAT3 signaling pathway. When cultured with CAF-CM, both p-JAK2 and p-STAT3 expression in lung cancer cells were increased, and total STAT3 expression remained unchanged. The expression of EMT-related gene E-cadherin was decreased, and vimentin was increased. The metastasis-related genes MMP2 and VEGF were also upregulated. This was consistent with the results when cells were treated with IL-6 (200 ng/ml) alone (Figure 4F).

**Figure 5:**
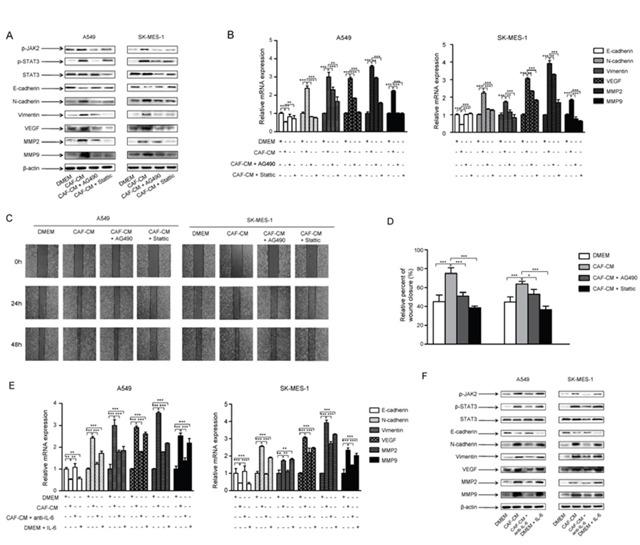
CAFs activated JAK2/STAT3 signaling in lung cancer cells Lung cancer cells were cultured with DMEM, CAF-CM, CAF-CM + JAK2 inhibitor AG490 (100 μM) or DMEM + STAT3 inhibitor STATIC (7.5 μM) for 24 h. **(A)** Protein expression were detected by Western blot. **(B)** mRNA expression were detected by qPCR. **(C)** Lung cancer cells were cultured with DMEM, CAF-CM, CAF-CM + JAK2 inhibitor AG490 (100 μM) or DMEM + STAT3 inhibitor STATIC (7.5 μM) for 24 to 48 h. Cell migration ability was measured by wound healing assays. **(D)** Relative adhension rates at 48 h time point were presented. **(E)** Lung cancer cells were cultured with DMEM, CAF-CM, CAF-CM + antiIL-6 or DMEM + IL-6 for 24h. mRNA expression were detected by qPCR. **(F)** Lung cancer cells were cultured with DMEM, CAF-CM, CAF-CM + antiIL-6 or DMEM + IL-6 for 24h. Protein expression were detected by Western blot. Values represent the mean ± SD from three independent experiments. *P<0.05; **P<0.01; ***P<0.001.

In order to confirm these results, one strategy we used was to block IL-6 by preincubating CAF-CM with 5 μg/ml of IL-6 neutralizing antibody for 4 h, then cultured with lung cancer cells. Another strategy was to inhibit JAK2 or STAT3 activation by pretreating lung cancer cells with JAK2 specific inhibitor AG490 (100 μM) or STAT3 specific inhibitor Stattic (7.5 μM) for 24 h, then cultured with CAF-CM. Our results showed that IL-6 neutralizing antibody, JAK2 inhibitor and STAT3 inhibitor could effectively reverse the effect of CAF-CM on the activation of JAK2/STAT3 signaling pathway (Figure 5A), and the regulation of EMT- and metastasis-related genes (Figure 5B).

To further evaluated the role of JAK2/STAT3 signaling pathway in the effect of CAF on cell migration ability, lung cancer cells were pretreated with JAK2 or STAT3 inhibitor, then cultured with CAF-CM. The wound healing results showed that the blockade of JAK2/STAT3 signaling effectively reversed the enhanced abilities of migration of lung cancer cells by CAF-CM (Figure 5C, 5D). Collectively, these findings indicated that CAFs induced EMT and enhanced the metastasis potential of lung cancer cells by activation of IL-6/STAT3 signaling pathway.

### CAFs enhanced tumor formation of human lung cancer cell lines

**Figure 6:**
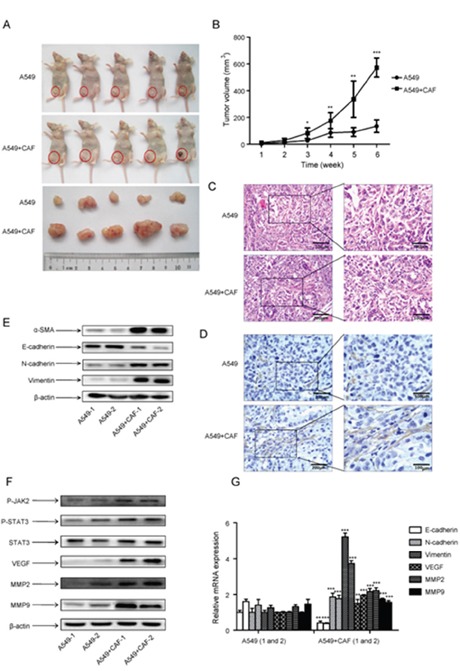
CAFs enhanced tumor formation and activated JAK2/STAT3 signaling in human lung cancer cells 3×10^6^ A549 cells, alone or with 9×10^6^ CAF (ratio1:3) were injected subcutaneously into the left lower flank of mice. Each treatment group consisted of 5 mice. **(A)** Tumor formation was inspected and tumor volume was measured after 6 weeks. **(B)** The average tumor volume of the two groups was measured at indicated times. **(C)** Tumor tissues were dissected and H&E staining was performed. **(D)** α-SMA expression in tumor tissues was detected by immunohistochemistry. **(E)** α-SMA expression in 2 tumor tissues from each group was detected by Western blot. **(F)** The protein expression in tumor tissues was detected by Western blot. **(G)** The mRNA expression in 2 tumor tissues from each group was detected by qPCR. Values represent the mean ± SD from three independent experiments. *P<0.05; **P<0.01; ***P<0.001.

As our *in vitro* study showed that CAFs stimulated lung cancer cell growth (Figure 2B), we next examined its effect on tumor formation *in vivo*. Lung cancer A549 cells alone or with human CAFs were subcutaneously injected into mice, and tumor volume were measured every week for 6 weeks period. The tumor growth was significantly faster in A549 cells with CAFs group than A549 cells alone group, and it was 3.5-fold faster than A549 cells alone group after 6 weeks (Figure 6A, 6B).

To determine the impact of CAFs on tumor growth, mice tumor specimens were obtained from mice injected with either cancer cells alone or co-injected with CAFs. H&E staining revealed that there was no difference between A549 cells with CAFs group and A549 cells alone group (Figure 6C). We also detected the expression of α-SMA by immunohistochemistry. Figure 6D showed that the amount of activated fibroblast with positive α-SMA staining in A549 cells with CAFs group was higher than in A549 alone group. Further Western blot results on mice tumor specimens also showed the expression of α-SMA was higher in A549 cells with CAFs group than A549 cells alone group (Figure 6E). We also found that the expression of EMT-related genes E-cadherin and N-cadherin were higher, whereas vimentin was lower in A549 cells with CAFs group than A549 cells alone group (Figure 6E, 6G). These results suggested that CAFs contributed to the tumor growth and induced EMT.

We then examined the JAK2/STAT3 pathway in tumor tissue. Both p-JAK2 and p-STAT3 level were significantly increased by CAFs compared with A549 cells alone, and total STAT3 level remained unchanged (Figure 6F). Furthermore, the expression of metastasis-related genes MMP2 and VEGF were increased in A549 cells with CAFs group at both mRNA and protein level (Figure 6F, 6G). Taken together, these results demonstrated that CAFs promoted tumor growth *in vivo* by inducing EMT and activating JAK2/STAT3 pathway.

## DISCUSSION

The tumor microenvironment plays a critical role in tumor progression and invasion. Tumor progression and metastasis does not solely depend on tumor cells, it is also controlled by tumor microenvironment. Tumor-localized CAFs may comprise up to more than half of the tumor mass, and there are multiple communications between CAFs and cancer cells [19]. In order to investigate the effect of CAFs on lung cancer metastasis, we isolated CAFs from lung cancer tumor tissues, and also NFs from adjacent normal tissues. Although there are several markers are used for CAFs identification, Kalluri reported that α-SMA and FAP are more specific [6]. We found that CAFs isolated from lung cancer tumor tissues expressed higher level of α-SMA and FAP than NFs, however, there was no significant difference in morphology between CAFs and NFs, these results is consistent with other groups’ study [19, 20].

We first examined the effect of CAFs on cell growth. Our results showed that CAF-CM stimulated lung cancer cell growth. Interestingly, when lung cancer cells were co-cultured with CAFs, Yasushi *et al* found that CAFs did not increase cancer cell proliferation [19]. There are studies showed that CAFs may promote or inhibit cancer cell proliferation, suggesting that the differential proliferative capacity of CAFs depends on the origin of fibroblast and cancer cell types [21–23] [24].

Metastasis is the common cause of death in cancer patients. To explore the effect of CAFs on lung cancer cell metastasis, we performed wound healing assay and transwell chamber assay. Our results demonstrated that CAFs enhanced lung cancer cell migration and invasion *in vitro*. EMT is a process by which epithelial cells lose their cell polarity and cell-cell adhesion, and gain migratory and invasive properties to become mesenchymal cells, it is a well-recognized process in initiating tumor cell invasion and metastasis [25, 26]. We found that CAFs induced EMT in lung cancer cells characterized by decreased E-cadherin level and increased vimentin level. Yu *et al* reported that CAFs induce EMT in breast cancer cells [27], Zhuang *et al*’ study showed that TGFβ secreted by CAFs induces EMT of bladder cancer cells [11]. In lung cancer, there are also studies indicating that CAFs induce EMT [19, 28]. These studies suggest that CAFs-induced EMT might be a common mechanism underlying the obtain of metastasis potential of cancer cells.

Since the invasion and metastasis of tumor is mediated by metastasis-related genes, we detected the effect of CAFs on expression of metastasis-related genes. Angiogenesis is the process of the formation of new blood vessels from pre-existing vessels, it is crucial for the growth and metastasis of tumors [29]. CAFs might promote angiogenesis of cancer cells to support tumor growth, invasion and metastasis [30, 31]. VEGF is a pro-angiogenic growth factor, playing an important role in tumor metastasis. Recently, VEGF/VEGF receptor pathway has become to be a promising target for tumor metastasis therapy [32, 33]. Matrix metalloproteinase (MMPs) are essential proteases involved in cancer invasion and metastasis. One of the key MMPs is MMP-2, which is highly expressed in tumor tissue. The modulation of MMP-2 effectively affects tumor metastasis [34, 35]. Our study showed that CAFs upregulated the expression of both VEGF and MMP-2, suggesting that CAFs promoted lung cancer cell metastasis by modulating metastasis-related genes.

CAFs secrete various factors into the tumor microenvironment to facility tumor cell malignancy, including chemokines and growth factors [30]. To further explore the underlying mechanism, we investigated the molecules and signaling pathways which mediated the promoting effect of CAFs on lung cancer cell metastasis potential. Numerous studies showed that IL-6 and STAT are involved in tumor progression and metastasis in various types of cancer [36, 37], including breast cancer [38], renal cancer [39], prostate cancer [40], and lung cancer [41, 42]. Recently, Silvestre *et al* demonstrated the role of IL-6/IL-6 receptor signaling in promoting growth of lung cancer cells in mouse model [43], Yeh *et al* found that estrogen receptor α in CAFs suppresses prostate cancer invasion via reducing IL-6 and CCL5 in the tumor microenvironment [44], and Jobe *et al* showed that simultaneous blocking of IL-6 and IL-8 is sufficient to inhibit CAFs-induced human melanoma cell invasiveness [45]. These studies drove us to investigate the role of IL-6/STAT signaling pathway. Our study, both *in vitro* and *in vivo*, demonstrated that CAFs activated IL-6/STAT3 signaling pathway, leading to the enhanced metastasis potential of lung cancer.

In summary, CAFs enhance lung cancer cell metastasis potential by inducing EMT programming and modulating metastasis-related genes. The mechanistic study demonstrates that IL-6/STAT3 signaling pathway mediates the enhanced effect of CAFs on lung cancer cells metastasis potential. Our findings suggests that the IL-6/STAT3 signaling pathway activated by CAFs could be a promising target for anti-cancer therapies.

## MATERIALS AND METHODS

### Reagents

Human recombinant IL-6 and IL-6 neutralizing antibody were purchased from R&D Systems Inc. (Minneapolis, MN, USA). STAT3 inhibitor Stattic was purchased from Sigma-Aldrich Inc. (St Louis, MO, USA), JAK2 inhibitor AG490 was purchased from Abcam (Cambridge, MA, USA).

Antibodies used for western blotting were mouse anti-E-cadherin (Invitrogen, Carlsbad, CA, USA), mouse anti-vimentin (BD Bioscience, San Jose, CA, USA), rabbit anti-phospho-JAK2 (Cell Signaling Technology, Beverly, MA, USA), rabbit anti-STAT3 (Cell Signaling Technology), rabbit anti-phospho-STAT3 (Cell Signaling Technology), mouse anti-α-SMA (Abcam), rabbit anti-MMP2 (Cell Signaling Technology), mouse anti-VEGF (Cell Signaling Technology), rabbit anti-TWIST (Cell Signaling Technology), mouse anti-β-actin (Sigma-Aldrich). The secondary antibodies coupled to HRP were purchased from ZSGB-BIO (Beijing, China)

### Cell culture

Human lung cancer cell lines A549, SK-MES-1 and H661 were purchased from American Type Culture Collection (Manassas, VA, USA). Cells were grown and maintained in DMEM medium (GIBCO BRL, Grand Island, NY, USA) at 37°C, 5% CO2. Medium was supplemented with 10% fetal bovine serum.

### Isolation and culture of stromal fibroblasts

Human cancer associated fibroblasts (CAFs) and normal lung fibroblasts (NFs) were isolated from primary tumor tissue and adjacent normal tissues. These tissues were obtained from 2 patients diagnosed as NSCLC at Tianjin Medical University General Hospital (TMUGH; Tianjin, China). These patients had not been treated with chemotherapy before surgery. The collection and the using of specimens were approved by the Institutional Review Board of TMUGH and the informed consent was obtained from all patients.

The fresh tissues were sliced and digested with 1 mg/mL collagenase A (Roche Applied Science, Mannheim, Germany) and 25 mg/mL Trypsin at 37°C for 40 min. Then the mixture was strained through a strainer and the cells were collected and cultured in Mc Cow’s 5A (GIBCO BRL) supplemented with 10% FBS, 1% L-glutamine, 1% Non-essential amino acids (Hyclone, Logan, UT, USA), 100 U/mL penicillin and 100 μg/mL streptomycin. After 2-3 passages, the cells were assessed by qPCR, Western blot and immunfluorescence staining for α-SMA and FAP, which were highly expressed in stromal fibroblasts, to confirm that a confluent and homogeneous monolayer of stromal fibroblasts was formed.

To prepare the conditioned medium (CM), CAFs and NFs were cultured for 72h, the conditioned medium were collected and centrifuged for 10 min at 3000 rpm to remove cell debris. All *in vitro* experiments were performed in triplicate using 2 pairs of CAFs and NFs which were at less than 10 passages.

### Cell proliferation assay

Cells were plated at a density of 3 × 10^3^ cells in triplicate in a 96-well plate. At 24h post-seeding, conditioned medium was added and cultured for 3 days, and the fresh medium was used as control. Cell proliferation were determined by the Cell Counting Kit-8 (Dojindo, Kumamoto, Japan) following the manufacture’s instruction.

### Wound healing assay

Cell migration was examined by wound healing assay as previously described [46]. Briefly, cells were seeded in six-well plates and cultured with different mediums. A clean wound area across the well was made by a pipette tip, and cells were allowed to migrate in the medium. Photographs were taken by a microscope (Nikon, Tokyo, Japan) at x40 magnification at an appropriate time to estimate the distance cells migrated.

### Cell invasion assay

Cell invasion ability was examined by trans-well assay as previously described [47]. To perform the invasion assay a 24-well transwell chamber (Costar, New York, NY, USA) with a polycarbonate membrane with a pore size of 8 μm was used. The membrane was coated with matrigel (BD Biosciences). 1 × 10^4^ cells pretreated with either CAF-CM or NF-CM for 48h were added to the upper compartment of the chamber, the lower chamber was filled with either CAF-CM or NF-CM. After cultured for 48 h in a 37°C, 5% CO_2_ atmosphere, non-invading cells on the upper surface of the membrane were removed by using a cotton swab; invading cells on the lower surface of the membrane were stained with 1% crystal violet and counted in 10 random fields from each membrane under a microscope at x200 magnification.

### Quantitative PCR

Total RNA was extracted from cells or tissues using Trizol (Invitrogen, Carlsbad, CA,USA). Reverse transcription was performed by using random primers in TaKaRa kit (Dalian, China) following manufacturer’s instruction. The expression of genes were measured by quantitative PCR (qPCR) using Power SYBR Green Master Mix (ABI, Foster City, CA, USA) on an ABI Prism 7900HT Sequence Detector System. All primers were designed by Primer 5.0 software and synthesized by SBS Genetech (Beijing, China). The primers sequences were listed in Table 1. The expression level of genes were calculated based on the cycle threshold (Ct) values. The results were calculated as 2^-ΔΔCt^ by comparing the Ct values of target genes with the Ct values of the reference gene (GAPDH).

**Table 1:** PCR primer sequences

Primers	Sequence (5’-3’)	Length of amplicons (bp)
*α-SMA*	Forward CACTGCCGCATCCTCATC	161
	Reverse TGCTGTTGTAGGTGGTTTCAT	
*FAP*	Forward GAAAGAAAGGTGCCAATA	116
	Reverse GATCAGTGCGTCCATCA	
*IL-6*	Forward AGACTTGCCTGGTGA	100
	Reverse GCTCTGGCTTGTTCC	
*E-cadherin*	Forward AAGGTGCTCTTCCAG	79
	Reverse GCGGCATTGTAGGTGT	
*Vimentin*	Forward TTCCTCCCTGAACCTGA	121
	Reverse AGTTTCGTTGATAACCTGTCC	
*VEGF*	Forward AGGAGGAGGGCAGAATCATCA	76
	Reverse CTCGATTGGATGGCAGTAGCT	
*MMP2*	Forward GCGGCGGTCACAGCTACTT	71
	Reverse CACGCTCTTCAGACTTTGGTTCT	
*GAPDH*	Forward TGCACCACCAACTGCTTAGC	87
	Reverse GGCATGGACTGTGGTCATGAG	

*α-SMA*, α-smooth muscle actin; *FAP*, fibroblast activation protein; *IL-6*, interleukin 6; *VEGF*, vascular endothelial growth factor; *MMP2*, matrix metallopeptidase 2; *GAPDH*, glyceraldehydes-3-phoshate dehydrogenase.

### Western blotting

Western blottings were performed as previously described [48]. Briefly, harvested cells were lysed with RIPA buffer containing protease inhibitor (Sigma-Aldrich). The proteins were separated by SDS-PAGE and transferred to a nitrocellulose membrane (Millipore, Bedford, MA, USA). The membranes were blocked with 5% non-fat milk in Tris-buffered saline containing 0.5% Tween-20 (TBS-T) for 1.5 h at room temperature, then probed with primary antibodies for α-SMA, p-JAK2, p-STAT3 (Tyr705), STAT3, E-cadherin, Vimentin, VEGF, MMP2 in TBS-T containing 5% BSA at 4°Covernight. After washing the membranes were probed with the HRP-conjugated secondary antibodies. The protein bands were developed with Chemiluminescent HRP Substrate (Millipore, Bedford, MA, USA)

### Enzyme-linked immunosorbent assay

A549 cells, SK-MES-1 cells, CAFs and NFs were cultured for indicated times, the supernatants were collected for IL-6 detection using an human IL-6 ELISA kit (eBioscience, San Diego, CA, USA), according to the manufacturer’s instructions. Briefly, immunoassay plates were precoated with the capture Antibody anti-Human IL-6 at 4°C overnight. After blocked for 1h, supernatants or standard human IL-6 were added and incubated for 2 h. The plates were washed and incubated with biotin labeled IL-6 antibody for 2 h, then with streptavidin-HRP complex. After 30 min incubation, tetramethylbenzidine (TMB) color-substrate solution was added and incubated for 15 min. The plates were read on a microplate reader (BioTek Instruments Inc., Winooski, VT, USA) at 450 nm.

### Immunofluorescence

Cells were seeded on the cover-slips in 24-well plates and cultured overnight, then fixed in 4% paraformaldehyde for 20 min. After washing with PBS, cells were permeabilised in 0.25% Triton X-100 for 15 min, washed with PBS and blocked with 5% BSA for 60 min. Then cells were incubated with primary antibody overnight at 4°C. After washing with PBS, cells were incubated with fluorescein isothiocyanate conjugated secondary antibody (Life Technology, Carlsbad, CA, USA) at 37°C for 40 min. Nuclei were stained with DAPI. The protein expression was observed and analyzed using a fluorescence microscope (Nikon, Tokyo, Japan,).

### Immunohistochemistry

Mice tissue specimens were formalin fixed and paraffin embedded. The specimens were incubated with primary antibody anti-α-SMA (1:500) at 4°C overnight. After washing with PBS the specimens were probed with secondary antibody, then stained with diaminobenzidine (DAB). Routine haematoxylin and eosin staining (H&E) was also performed.

### Mouse tumor models

BALB/c nude mice were purchased from the Cancer Institute of the Chinese Academy of Medical Science (Beijing, China). Xenograft experiments were carried out in accordance with the Tianjin Medical University Institutional Animal Care and Use Committee guidelines. Each treatment group consisted of 5 mice. 3×10^6^ A549 cells, alone or with 9×10^6^ CAF (ratio1:3), were resuspended in 100 μl PBS and injected subcutaneously into the left lower flank of mice. Beginning 1 week after injection, tumor dimensions were measured every week, and tumor volume was calculated as: volume (mm^3^) = d^2^ × D/2, where the d and D were the shortest and the longest diameters. The experiment had been performed for 6 weeks. Tumor tissues were dissected and used for immunohistochemistry, and mRNA and protein extraction.

### Statistical analysis

The data were presented as mean ± standard deviation (S.D.). Variance analysis between groups was performed by one-way ANOVA. Dunnett multiple comparison test was used to compare the difference between control and treatment groups. Statistical significance was defined as p < 0.05.
